# Management of patients with lower-risk myelodysplastic syndromes

**DOI:** 10.1038/s41408-022-00765-8

**Published:** 2022-12-14

**Authors:** Andrew M. Brunner, Heather A. Leitch, Arjan A. van de Loosdrecht, Nicolas Bonadies

**Affiliations:** 1 Massachusetts General Hospital, Boston, MA USA; 2 Hematology, St. Paul’s Hospital, University of British Columbia, Vancouver, BC Canada; 3 Department of Hematology, Cancer Center Amsterdam, Amsterdam University Medical Center, VU University Amsterdam, Amsterdam, Netherlands; 4 Department of Hematology and Central Hematology Laboratory, Inselspital, Bern University Hospital, University of Bern, Bern, Switzerland

**Keywords:** Haematological diseases, Therapeutics, Diagnosis, Prognosis

## Abstract

Myelodysplastic syndromes (MDS) are a heterogeneous group of hematopoietic stem cell disorders characterized by ineffective hematopoiesis with abnormal blood cell development (dysplasia) leading to cytopenias and an increased risk for progression to acute myeloid leukemia (AML). Patients with MDS can generally be classified as lower- (LR-MDS) or higher-risk (HR-MDS). As treatment goals for patients with LR-MDS and those with HR-MDS differ significantly, appropriate diagnosis, classification, and follow-up are critical for correct disease management. In this review, we focus on the diagnosis, prognosis, and treatment options, as well as the prediction of the disease course and monitoring of treatment response in patients with LR-MDS. We discuss how next-generation sequencing, increasing knowledge on mechanisms of MDS pathogenesis, and novel therapies may change the current treatment landscape in LR-MDS and why structured assessments of responses, toxicities, and patient-reported outcomes should be incorporated into routine clinical practice.

## Introduction

Myelodysplastic syndromes (MDS) comprise a heterogeneous group of hematopoietic stem and progenitor cell disorders characterized by ineffective hematopoiesis leading to dysplasia, cytopenias, and an increased risk of evolution to acute myeloid leukemia (AML) [1, 2]. MDS occurs in all age groups but mainly affects the elderly, with a median age of onset above 70 years [3, 4]. The majority of MDS diagnoses are “lower-risk” diseases (LR-MDS), indicating a relatively lower risk of death or progression to AML in the immediate period after diagnosis [5, 6]. However, the presence of anemia and complications related to cytopenias, transfusions, and inflammation can negatively affect comorbid conditions, potentially reducing the quality of life (QoL) and increasing the mortality of these patients relative to the general population [7, 8]. Molecular sub-characterization of MDS has emerged following the discovery of recurrent somatic driver mutations [9], though understanding of the mechanisms involved in clonal evolution and its impact on disease phenotype remains incomplete [10]. Together with the emergence of effective therapies for LR-MDS targeting disease-associated pathways and processes, e.g., involving transforming growth factor beta (TGF-β) signaling, DNA methylation, and other epigenetic targets, our understanding of the LR-MDS pathogenesis also advances [11].

Here we review the diagnosis, prognosis, and treatment—including treatment response monitoring—of patients with LR-MDS. We discuss emerging therapies and why structured assessments of responses, toxicities, and patient-reported outcomes (PROs) should be incorporated into guidelines and recommendations for daily clinical practice to improve clinical outcomes.

### Diagnosis

Rapid and accurate diagnosis of MDS remains critical, and two new classification systems were recently proposed [12, 13]. The initial MDS assessment should provide data regarding disease prognostication and should inform about appropriate treatment choices. MDS diagnosis is based on the quantitative and qualitative assessment of a peripheral blood smear; bone marrow cytology and histology; cytogenetic and mutational analyses; and flow cytometry immunophenotyping [14]. Patients with suspected MDS should undergo a detailed medical history check on exposure to genotoxic agents (e.g., chemotherapy, therapeutic radiation, or organic solvents [e.g., benzene]). We also recommend assessing family history for potential signs of germline predisposition and constitutional stigmata (e.g., findings suggestive of telomere disease) and testing of germline tissue obtained through fibroblast cultures, when required. In addition, comprehensive molecular testing may identify patients with later onset germline mutations such as *DDX41*, some mutations in telomere disease, or *RUNX1*. This information should then be integrated with laboratory analyses (e.g., blood counts, peripheral blood smear, bone marrow aspirate/biopsy, cytogenetics, including a full karyotype, flow cytometry immunophenotyping, and mutational analysis) to exclude other conditions [15–18]. The International Working Group (IWG) for flow cytometry in MDS (IMDS Flow) of the MDS European LeukemiaNet (ELN) published guidelines for multi-parameter flow cytometry immunophenotyping in MDS outlining markers of particular interest [18–21] (Table S1). Finally, anemia symptoms, fatigue, bleeding, infections, and inflammation should be carefully assessed and checked during treatment. Numerous MDS diagnostic guidelines are available from several consortia, including the MDS ELN, European Society for Medical Oncology (ESMO), National Comprehensive Cancer Network^®^ (NCCN^®^), and MDS-Right group [6, 15, 16, 22].

**Fig. 1:**
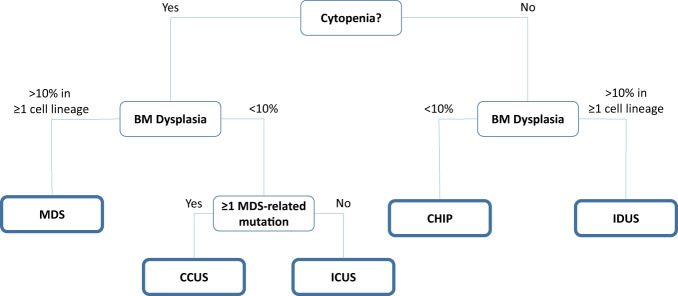
Diagnostic algorithm for MDS, ICUS, IDUS, CHIP, and CCUS [9, 14, 16, 30, 36]. Abbreviations: *BM* bone marrow, *CCUS* clonal cytopenias of uncertain significance, *CHIP* clonal hematopoiesis of indeterminate potential, *ICUS* idiopathic cytopenias of uncertain significance, *IDUS* idiopathic dysplasia of unknown significance, *MDS* myelodysplastic syndromes.

Until recently, the 2017 4th revision of the World Health Organization (WHO) classification of myeloid neoplasms and acute leukemia provided guidelines for the diagnosis and subclassification of MDS (Table 1) [23] using an integrated approach based on clinical, hematologic, morphologic, genetic, flow cytometric, and molecular findings. A complete karyotype remains important in the diagnosis and prognosis of MDS; when conventional cytogenetics testing fails, fluorescence in situ hybridization (FISH) probes, for instance, of chromosomes 5, 7, 8, 17, and 20, can be useful in the prognostication and for monitoring disease response after treatment [24]. Subsequent data has underscored the importance of specific mutations in disease presentation and prognosis. Importantly, WHO classification may correlate with disease risk, but typically other prognostic tools are used for risk assessment (e.g., International Prognostic Scoring System [IPSS], Revised IPSS [IPSS-R], or molecular IPSS [IPSS-M]). For instance, some data suggest that *SF3B1*-mutant MDS, characterized by ring sideroblasts (RS), ineffective erythropoiesis, and an indolent clinical course, should be recognized as a distinct nosologic entity (Table 1) [23, 25]. This growing understanding of the molecular pathogenesis of disease has also identified overlapping features between MDS and other clonal marrow processes. This is particularly challenging in the era of molecular diagnostics, where clonal abnormalities may exist in the absence of MDS-defining dysplasia or cytogenetic criteria. Therefore, immunophenotypic or molecular alterations indicative of clonality have been recently introduced into the minimal diagnostic criteria for MDS in situations where morphological findings are insufficient (Fig. 1; Table S2) [23, 26]. These additions aim to support clinicians in finding the precise diagnosis for cases with inconclusive morphological and cytogenetic alterations [23, 26]. In patients with clonal hematopoiesis as well as cytopenias, distinguishing between clonally driven cytopenias and secondary causes of low blood counts remains challenging [26]. It is particularly important for patients with suspected LR-MDS, to distinguish MDS-related cytopenias from other causes of cytopenias presenting on top of background clonal hematopoiesis, including aplastic anemia, paroxysmal nocturnal hemoglobinuria, nutritional deficiencies, autoimmune disorders, and infections [9]. For instance, RS formation, which may masquerade as MDS, can also be associated with copper deficiency and alcohol dependency [27, 28]. Another condition that may be associated with MDS is the VEXAS syndrome, characterized by fever, inflammation, and vacuoles in hematopoietic cells and related to a mutation in the *UBA1* gene [29].

**Table 1:** Key classification criteria for MDS according to WHO 2017, WHO 2022, and ICC 2022.

Entity name	Key diagnostic characteristics
*WHO 2017 classification (4th edition)* [23]	
MDS with single lineage dysplasia (MDS-SLD)	• Dysplasia in ≥10% of RBCs or WBCs or MKs
MDS with multilineage dysplasia (MDS-MLD)	• Dysplasia in ≥10% of 2 or 3 of RBCs or WBCs or MKs
MDS with ring sideroblasts (MDS-RS)	
MDS-RS with single lineage dysplasia (MDS-RS-SLD)	• Dysplasia in ≥10% of RBCs or WBCs or MKs • RS in ≥15% of nucleated erythroid cells, or in ≥5% of nucleated erythroid cells in the presence of *SF3B1* mutation
MDS-RS with multilineage dysplasia (MDS-RS-MLD)	• Dysplasia in ≥10% of 2 or 3 of RBCs or WBCs or MKs • RS in ≥15% of marrow erythroid elements, or in ≥5% of marrow erythroid elements with *SF3B1* mutation
MDS with excess blasts (MDS-EB)	
MDS-EB1	• 5–9% blasts in BM or 2–4% in PB
MDS-EB2	• 10–19% blasts in BM or 5–19% in PB
MDS with isolated del(5q)	• Deletion of chromosome 5q, either alone or with 1 additional abnormality except −7 or del(7q)
SF3B1-mutated MDS (proposed new classification)	• Cytopenia defined by standard hematologic values • Somatic *SF3B1* mutation • Isolated erythroid or multilineage dysplasia^a^ • BM blasts <5% and PB blasts <1% • WHO criteria for MDS with isolated del(5q), MDS/MPN-RS-T or other MDS/MPNs, and primary myelofibrosis or other MPNs are not met • Normal karyotype or any cytogenetic abnormality other than del(5q); monosomy 7; inv(3) or abnormal 3q26, complex (≥3) • Any additional somatically mutated gene other than *RUNX1* and/or *EZH2*^b^
*WHO 2022 classification (5th edition)* [12]	
MDS, morphologically defined	
MDS with low blasts (MDS-LB)	• <5% blasts in BM or <2% in PB
MDS, hypoplastic (MDS-h)	• <5% blasts in BM or <2% in PB • By definition, ≤25% BM cellularity, age adjusted
MDS with increased blasts (MDS-IB)	
MDS-IB1	• 5–9% blasts in BM or • 2–4% in PB
MDS-IB2	• 10–19% blasts in BM or 5–19% in PB or Auer rods
MDS with fibrosis (MDS-f)	• 5–19% blasts in BM • 2–19% in PB
MDS with defining genetic abnormalities	
MDS with low blasts and isolated 5q deletion (MDS-5q)	• <5% blasts in BM or <2% in PB • 5q deletion alone, or with 1 other abnormality other than monosomy 7 or 7q deletion
MDS with low blasts and SF3B1 mutation (MDS-SF3B1)	• <5% blasts in BM or <2% in PB • Absence of 5q deletion, monosomy 7, or complex karyotype • *SF3B1* mutation • Detection of ≥15% RS may substitute for *SF3B1* mutation. Acceptable related terminology: MDS-LB and RS
MDS with biallelic TP53 inactivation (MDS-biTP53)	• <20% blasts in BM or in PB • Usually complex cytogenetics • Two or more *TP53* mutations, or 1 mutation with evidence of *TP53* copy number loss or cnLOH
*ICC 2022 classification* [13]	
MDS, not otherwise specified (NOS)	
MDS, NOS without dysplasia	• ≥1 cytopenia • <5% blasts in BM or <2% in PB^c^ • −7/del(7q) or complex cytogenetics • Any mutations, except multi-hit *TP53* or *SF3B1* (≥10% VAF)
MDS, NOS with single lineage dysplasia	• 1 dysplastic lineage • ≥1 cytopenias • <5% blasts in BM or <2% in PB^c^ • Any cytogenetics, except not meeting criteria for MDS-del(5q) • Any mutations, except multi-hit TP53; not meeting criteria for MDS-*SF3B1*
MDS, NOS with multilineage dysplasia	• ≥2 dysplastic lineage • ≥1 cytopenia • <5% blasts in BM or <2% in PB^c^ • Any cytogenetics, except not meeting criteria for MDS-del(5q) • Any mutations, except multi-hit *TP53*; not meeting criteria for MDS-*SF3B1*
MDS with excess blasts (MDS-EB)	• Typically ≥1 dysplastic lineage (not required) • ≥1 cytopenia • 5–9% blasts in BM or 2–9% in PB^c^ • Any cytogenetics • Any mutations, except multi-hit *TP53*
MDS/AML	• Typically ≥1 dysplastic lineage • ≥1 cytopenia • 10–19% blasts in BM or in PB^d^ • Any cytogenetics, except AML-defining • Any mutations, except *NPM1*, bZIP *CEBPA*, or *TP53*
MDS with del(5q) [MDS-del(5q)]	• Typically ≥1 dysplastic lineages (not required) • ≥1 cytopenia • Thrombocytosis allowed • <5% blasts in BM or <2% in PB^c^ • del(5q), with up to 1 additional, except −7/del(7q) • Any mutations, except multi-hit *TP53*
MDS with mutated *SF3B1* (MDS-*SF3B1*)	• Typically ≥1 dysplastic lineages (not required) • ≥1 cytopenia • <5% blasts in BM or <2% in PB • Any, except isolated del(5q), −7/del(7q), abn3q26.2, or complex *SF3B1* (≥10% VAF), without multi-hit *TP53*, or *RUNX1*
MDS with mutated TP53	• Any cytopenia • 0–9% BM and PB blasts • Multi-hit *TP53* mutation, or *TP53* mutation (VAF > 10%) and complex karyotype often with loss of 17p

^a^RS are not required for the diagnosis.

^b^Additional *JAK2V617F*, *CALR*, *or MPL* mutations strongly support the diagnosis of MDS/MPN-RS-T.

^c^Although 2% PB blasts mandates the classification of an MDS case as MDS-EB, the presence of 1% PB blasts confirmed on two separate occasions also qualifies for MDS-EB.

^d^For pediatric patients (<18 years), the blast thresholds for MDS-EB are 5–19% in BM and 2–19% in PB, and the entity MDS/AML does not apply.

Abbreviations: *AML* acute myeloid leukemia*, BM* bone marrow, *ICC* International Consensus Classification, *MDS* myelodysplastic syndromes, *MK* megakaryocyte, *MPN-RS-T* myeloproliferative neoplasm with RS and thrombocytosis, *PB* peripheral blood, *RBC* red blood cell, *RS* ring sideroblasts, *VAF* variant allele frequency, *WBC* white blood cell, *WHO* World Health Organization.

Recently, two updated classifications were published: the 5th edition of the WHO classification and the International Consensus Classification (ICC) of myeloid neoplasms and acute leukemias (Table 1) [12, 13]. There are minor differences between the classifications, which include the nomenclature of some MDS subgroups, minor variations in diagnostic thresholds, and several new diagnostic entities (Table 2). The ICC 2022 proposes categorizing MDS with single lineage dysplasia (MDS-SLD) and MDS with multilineage dysplasia (MDS-MLD), per the WHO 2017 revision, as MDS, not otherwise specified (MDS, NOS) with SLD or with MLD [13]. It also introduced a new MDS/AML category, defined as a cytopenic myeloid neoplasm with 10–19% blasts in peripheral blood or bone marrow, allowing patients to qualify for both MDS and AML clinical trials. The WHO 2022 revision replaced the term myelodysplastic syndromes with myelodysplastic neoplasms (still abbreviated as MDS) and regrouped MDS entities as MDS with defined genetic abnormalities and morphologically defined MDS [12]. It also categorizes MDS-SLD and MDS-MLD into a new category (MDS with low blasts; MDS-LB) and recognizes hypoplastic MDS (MDS-h) with <25% cellularity as a distinct entity. Both classifications replaced the MDS-RS category with the MDS with *SF3B1* category [12, 13], however, the WHO 2022 classification also permits the use of the term MDS with low blasts and RS, if wildtype *SF3B1* and ≥15% RS are present [12]. Both classifications include MDS-*TP53* as a separate entity, recognizing the generally poor outcomes in this molecular subset, while MDS-del(5q) remains the same. Notably, the MDS unspecified category from the WHO 2017 revision no longer exists in either 2022 classification system, as all subtypes now fit into one of the categories [12, 13]. The impact of the differences between the WHO and ICC 2022 classifications on clinical practice is not yet clear; for instance, the MDS-del(5q) and MDS with *SF3B1* entities remain identical, and the ICC 2022 MDS/AML category overlaps with the WHO 2022 MDS with increased blasts (MDS-IB) entity and is similar to the MDS with excess blasts (MDS-EB) category from the previous WHO edition. In general, prognostic models such as the IPSS, IPSS-R, or IPSS-M continue to guide clinical decision-making.

**Table 2:** Overview of similarities and differences in the WHO 2022 and ICC 2022 MDS criteria.

Feature	WHO 2022 [12]	ICC 2022 [13]
MDS with low blasts (MDS-LB)	Similarities • Entities MDS with low blasts (WHO) and MDS, NOS without dysplasia, MDS, NOS-SLD or MDS, NOS-SLD (ICC) are similar, but nomenclature differs	Similarities • Entities MDS with low blasts (WHO) and MDS, NOS without dysplasia, MDS, NOS-SLD or MDS, NOS-SLD (ICC) are similar, but nomenclature differs
	Differences • Has an MDS, hypoplastic entity	Differences • No MDS, hypoplastic entity
MDS with ring sideroblasts (MDS-RS)	Similarities • Has an MDS-*SF3B1* entity	Similarities • Has an MDS-*SF3B1* entity
	Differences • Retained the category MDS with low blasts and RS if no *SF3B1* mutation	Differences • If no *SF3B1* mutation, MDS will be classified as MDS, NOS-SLD/MLD
MDS with genetic abnormalities (*TP53*, *SF3B1*, del(5q))	Similarities • Has an MDS-*TP53* entity • Has an MDS-*SF3B1* entity • Has an MDS-del(5q) entity	Similarities • Has an MDS-*TP53* entity • Has an MDS-*SF3B1* entity • Has an MDS-del(5q) entity
	Differences • Biallelic MDS-*TP53* entity	Differences • MDS-*TP53* entity allows single hit mutation and a complex karyotype • MDS-*SF3B1* entity without *RUNX1* and abn3q26.2
MDS with excess blasts (MDS-EB)	Similarities • Has an AML (≥20% BM / PB) entity	Similarities • Has an AML (≥20% BM / PB) entity
	Differences • Cut-offs and nomenclature differ ∘ MDS-IB1 (5–9% BM / 2–4% PB) ∘ MDS-IB2 (10–19% BM / 5–19% PB) ∘ AML (≥10% BM / PB and AML-defining genetics) • Has an MDS with fibrosis entity	Differences • Cut-offs and nomenclature differ ∘ MDS-EB (5–9% BM / 2–9% PB) ∘ MDS / AML (10–19% BM / 10–19% PB) (new category only in ICC) ▪ Allows single *TP53* mutation for MDS / AML ∘ AML (Any BM / PB and AML-defining genetics) • No MDS with fibrosis entity

Abbreviations: *AML* acute myeloid leukemia*, BM* bone marrow, *ICC* International Consensus Classification, *MDS* myelodysplastic syndromes, *MDS-IB* MDS with increased blasts*, NOS-MLD* not otherwise specified with multilineage dysplasia, *NOS-SLD* not otherwise specified with single lineage dysplasia, *PB* peripheral blood, *RS* ring sideroblasts, *WHO* World Health Organization.

### Relevance of next-generation sequencing (NGS) and discrimination of pre-MDS conditions

Identification of somatic gene mutations and establishment of comprehensive mutational profiles of MDS samples using next-generation sequencing (NGS) plays a growing role in the diagnosis, prognosis, treatment selection, and monitoring of MDS [11, 30]. Importantly, relevant mutations affecting processes such as DNA methylation, pre-mRNA splicing, chromatin modification, transcription, and cell signaling may inform the development of new therapies. Clonal heterogeneity and its progressive evolution characterize many myeloid malignancies [31–33]. The presence of a clonal population at a median variant allelic fraction (VAF) of ~10%, can be identified in ~10% of adults aged >70 years with otherwise normal blood counts, and in up to 30% of those aged >80 years [34]; a phenomenon termed clonal hematopoiesis of indeterminate potential (CHIP) (Table S2) [16, 35]. These patients have a higher risk of subsequent hematologic malignancy and reduced overall survival (OS) compared with individuals without detectable mutations and a higher risk for adverse cardiovascular events and other degenerative-inflammatory age-associated disorders [34–37]. Clonal cytopenia of undetermined significance (CCUS; cytopenias with clonal mutation, but not meeting MDS diagnostic criteria), idiopathic cytopenia of uncertain significance (ICUS; cytopenias without a clonal mutation detected), and idiopathic dysplasia of unknown significance (IDUS; bone marrow dysplasia without a clonal mutation) have been described as “pre-MDS” conditions (Table S2) [16, 25, 35]. The risk of progression in patients with these forms of pre-MDS varies and is lower in those without identified evidence of clonal expansion, although ongoing prospective studies (e.g., SEARCH consortium) may better define this risk [16, 25, 35, 38].

### Risk stratification

To assess disease severity and treatment eligibility, patients with MDS are generally stratified by both disease- and patient-based risk. The most common risk-scoring systems are the IPSS and IPSS-R [5, 39]. The IPSS scoring system classifies patients into four risk categories: Low, Intermediate-1, Intermediate-2, and High, based on the number of cell lineages affected by cytopenias, blast percentages, and cytogenetic alterations [39]. The IPSS-R scoring system places greater emphasis on the impact of cytogenetic risk and bone marrow blast percentage and defines five risk categories: Very low, Low, Intermediate, High, and Very high (Table 3) [5]. The IPSS score is still considered for patient allocation to treatment, as most clinical trials for current MDS treatments have relied on IPSS classification [17]. Currently, patients are stratified into having either LR-MDS (IPSS-R categories: Very low-, Low-, or Intermediate-risk with a score of ≤3.5 points), with treatment focused on improving symptomatic cytopenias, or HR-MDS (Intermediate-risk category with a score of >3.5 points, and High-, or Very high-risk categories), with treatment focused on prolonging survival and delaying AML progression [40]. Likewise, patient-specific characteristics (i.e., patient age, presence of comorbidities, performance status, and frailty [reduced physical fitness]) have prognostic relevance in evaluating treatment-related mortality and hence treatment selection, including allogeneic hematopoietic stem cell transplantation (HSCT) [8, 41].

**Table 3:** IPSS-R and IPSS-M prognostic scoring and median OS by risk categories.

	IPSS-R [5]	Score	Median OS, years
Cytogenetics	• Very good: –Y or del(11q) • Good: normal, del(5q), del(12p), del(20q), double including del(5q) • Intermediate: del(7q), +8, +19, i(17q), any other single or double independent clones • Poor: –7, inv(3)/t(3q)/del(3q), double including –7/del(7q), 3 abnormalities • Very poor: >3 abnormalities	+0 +1 +2 +3 +4	–
BM blasts	• <2% • 2 to <5% • 5–10% • >10%	+0 +1 +2 +3	–
Hemoglobin	• ≥10 g/dL • 8 to <10 g/dL • <8 g/dL	+0 +1 +1.5	–
Platelets	• ≥100 × 10^9^/L • 50 to <100 × 10^9^/L • <50 × 10^9^/L	+0 +0.5 +1	–
ANC	• ≥0.8 × 10^9^/L • <0.8 × 10^9^/L	+0 +0.5	–
Category	• Very low • Low • Intermediate • High • Very high	0–1.5 2–3 3.5–4.5 5–6 7–10	8.8 5.3 3.0 1.6 0.8

IPSS-M score construction (adjusted Cox multivariable regression for leukemia-free survival) [42]			
Category and variable	Adjusted hazard ratio (95% CI)^a^	Model weight^b^	Median OS, years (25–75% range)
Clinical			–
BM blasts (%)	1.07 (1.05–1.09)	0.0704	–
Min (Platelets, 250 × 10^9^/L)	0.998 (0.997–0.999)	−0.00222	–
Hemoglobin (g/dL)	0.84 (0.81–0.88)	−0.171	–
Cytogenetics			
IPSS-R cytogenetic category (see above)	1.33 (1.21–1.47)	0.287	–
Gene main effects (17 variables, 16 genes)^c^			
*TP53*^*multihit*^	3.27 (2.38–4.48)	1.18	
*MLL*^*PTD*^	2.22 (1.49–3.32)	0.798	
*FLT3*^*ITDflTKD*^	2.22 (1.11–4.45)	0.798	
*SF3B1*^*5q*^	1.66 (1.03–2.66)	0.504	
*NPM1*	1.54 (0.78–3.02)	0.430	
*RUNX1*	1.53 (1.23–1.89)	0.423	
*NRAS*	1.52 (1.05–2.20)	0.417	
*ETV6*	1.48 (0.98–2.23)	0.391	
*IDH2*	1.46 (1.05–2.02)	0.379	
*CBL*	1.34 (0.99–1.82)	0.295	
*EZH2*	1.31 (0.98–1.75)	0.270	
*U2AF1*	1.28 (1.01–1.61)	0.247	
*SRSF2*	1.27 (1.03–1.56)	0.239	
*DNMT3A*	1.25 (1.02–1.53)	0.221	
* ASXL1*	1.24 (1.02–1.51)	0.213	
*KRAS*	1.22 (0.84–1.77)	0.202	
*SF3B1*^*α*^	0.92 (0.74–1.16)	−0.0794	
Gene residuals (1 variable, 15 genes; possible values of 0, 1, or 2)^d^			
Min (Nres,2)	1.26 (1.12–1.42)	0.231	
Category	Very low Low Moderate low Moderate high High Very high	≤−1.5 >−1.5 to −0.5 >−0.5 to 0 >0 to 0.5 >0.5 to 1.5 >1.5	10.6 (5.1–17.4) 6.0 (3.0–12.8) 4.6 (2.0–7.4) 2.8 (1.2–5.5) 1.7 (1.0–3.4) 1.0 (0.5–1.8)

^a^Hazard ratio is for the risk of leukemic transformation or death, adjusted for age, sex, and secondary/therapy related versus primary MDS. Cox regression was performed for 2428 patients with available covariables and leukemia-free survival data.

^b^Model weights were derived from the logarithm of the raw hazard ratios up to three significant digits. The following formula applies: IPSS-M score = 1.15467 + (*Σ*_*variables*__*j*_*w*_*j*_*x*_*j*_)/log(2), where *w*_*j*_ denotes the weight of variable *j*, and *x*_*j*_ the value of the variable *j* observed in a given patient.

^c^*SF3B1*^*5q*^ is the *SF3B1* mutation in the presence of isolated del(5q), i.e., del(5q) only or with one additional aberration, excluding –7/del(7q). *SF3B1*^*α*^ is the *SF3B1* mutation without co-mutations in *BCOR, BCORL1*, *RUNX1*, *NRAS*, *STAG2*, *SRSF2*, and del(5q).

^d^Nres is defined as the number of mutated genes within the following list: *BCOR*, *BCORL1*, *CEBPA*, *ETNK1*, *GATA2*, *GNB1*, *IDH1*, *NF1*, *PHF6*, *PPM1D*, *PRPF8*, *PTPN11*, *SETBP1*, *STAG2*, *WT1*. The variable min (Nres, 2) can therefore take the value 0, 1, or 2.

Abbreviations: *ANC* absolute neutrophil count, *BM* bone marrow, *CI* confidence interval, *IPSS-M* molecular International Prognostic Scoring System, *IPSS-R* Revised International Prognostic Scoring System, *MDS* myelodysplastic syndromes, *OS* overall survival.

Recently, the IPSS-M scoring system was introduced [42]. This model includes similar clinical, morphological, and cytogenetic parameters as IPSS-R, with additional genetic parameters (16 main effect genes and 15 residual genes) to classify patients into six risk categories: Very low, Low, Moderate low, Moderate high, High, and Very high. It outlines the recommended gene selection, sequencing, and analysis that allowed the identification of mutations present in 31 genes that, together with cytogenetic parameters, improve prognostic discrimination of patients compared with the IPSS-R model [42]. In practice, we assess these mutations to a level of 1–5% VAF, with consideration of larger NGS panels that may also assess other relevant mutations, such as *DDX41*. Importantly, some mutations (*TP53*^*multihit*^*, FLT3, KMT2A [MLL*^*PTD*^*]*) provide additional adverse prognostic risk, while others may suggest a more favorable disease course (*SF3B1*), though the outcome may be modulated by co-mutation patterns (Table 3) [42]. Although molecular features are increasingly involved in prognosis, it is important to consider how these can be intertwined with morphology; for instance, how the favorable association with *SF3B1* mutations may not add independent prognostic value after accounting for RS—like in the case of the WHO 2017 categories of refractory anemia with RS (RARS) or refractory cytopenia with multilineage dysplasia and RS (RCMD-RS) [43, 44]. Of note, the IPSS-M model includes patients with therapy-related MDS (t-MDS), which arises following cytotoxic chemotherapy and radiation treatment of a neoplastic or non-neoplastic disorder, or both [42]. Patients with t-MDS have previously been categorized within the WHO classification system as having a type of therapy-related myeloid neoplasm, alongside patients with therapy-related AML (t-AML) and t-MDS/myeloproliferative neoplasms (MPN), and historically were considered to have universally poor outcomes. However, the IPSS-M was able to stratify them into different risk groups, suggesting that molecular drivers of the disease may improve risk assessment more than clinical history alone [42].

## The general approach to the management of patients with LR-MDS

**Fig. 2:**
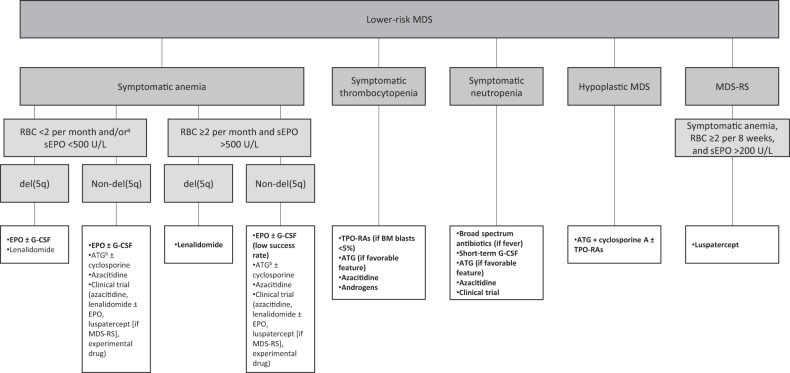
Current treatment options for lower-risk MDS. Bold text indicates first-line therapy. ^a^ESMO 2014 [45]: RBC <2 per month or sEPO <500 U/L. ^b^ESMO 2014 [45]: if age <60–65 years and favorable features (including hypoplastic BM, blasts <5%, normal karyotype, HLA-DR15-positivity, younger age [<60 years], lower risk according to IPSS [10, 95]) for response to ATG; ESMO 2021 [16]: if age <65–70 years and favorable features for response to ATG. Abbreviations: *ATG* anti-thymocyte globulin, *BM* bone marrow, *EPO* erythropoietin, *ESMO* European Society for Medical Oncology, *G-CSF* granulocyte colony-stimulating factor, *IPSS* International Prognostic Scoring System, *MDS* myelodysplastic syndromes, *MDS-RS* myelodysplastic syndrome with ring sideroblasts, *sEPO* serum erythropoietin, *TPO-RA* thrombopoietin-receptor agonist.

Following an appropriate MDS diagnosis and risk stratification, treatment is tailored toward the individual patient [10]. Most patients with LR-MDS will live with malignant hematopoiesis for many years, therefore, treatment goals focus on the improvement of disease-related symptoms and QoL. This is usually related to the management of cytopenias, most commonly anemia, and managing sequelae of disease and therapy (e.g., iron overload) [6, 15, 16, 22]. We, therefore, develop a disease management plan and treatment sequence with this in mind. Continuous development of diagnostics, therapies, and improving knowledge of MDS pathogenesis contribute to the evolving MDS management recommendations (e.g., ESMO [16, 45], ELN [15], NCCN^®^ [22], and the MDS Europe platform [46]). Figure 2 outlines LR-MDS management recommendations based on ESMO guidelines [16, 45]. A recent study proposed 29 guideline-based indicators, defined as measurable elements in the areas of diagnosis, therapy, and care provider infrastructure, for the assessment of the quality of care, which is currently undergoing validation [17]. Nonetheless, such efforts underscore the importance of including patient-centered outcomes in MDS management.

### Currently approved treatments for LR-MDS

The most common complication of LR-MDS is progressive anemia, which eventually leads to a requirement for regular red blood cell (RBC) transfusions [6, 47]. We administer erythropoiesis-stimulating agents (ESAs), which increase RBC production in the bone marrow, as first-line therapy for patients with LR-MDS and symptomatic anemia. There is, however, a significant variation in response quality (30–60%) and duration (1–2 years) with ESA use. Furthermore, as ESAs are not curative, eventually, patients will stop responding to therapy. Patients with low RBC transfusion requirement and serum erythropoietin (sEPO) below 200–500 mU/mL may be more likely to respond to ESAs, whereas those with high RBC transfusion requirement or high sEPO >500 mU/mL have a lower chance (<10%) of achieving a response [15, 16, 45]. A recent analysis of the EUMDS Registry study showed that patients with LR-MDS who received ESAs at the onset of anemia, but before starting RBC transfusion therapy, had improved survival, therefore supporting the consideration of early ESA treatment and further prospective validation of optimal ESA timing [48].

For some MDS subgroups, we consider other therapies in the frontline setting. The NCCN Clinical Practice Guidelines in Oncology (NCCN Guidelines^®^) suggest that lenalidomide may be utilized as first- or second-line therapy for patients with MDS-del(5q) (typically ±1 other cytogenetic abnormality, excluding those involving chromosome 7) and transfusion dependency, or as second-line treatment after ESA failure. The NCCN Guidelines^®^ also allow for a first-line trial of ESAs if desired [22], while ELN guidelines suggest a trial with growth factors before initiating lenalidomide [15]. Post hoc data show no differences in QoL with lenalidomide [49], and recent real-world studies in MDS-del(5q) have demonstrated long-term responses (from 21 to 32 months) and alleviation of anemia [50, 51]; nevertheless, further studies are needed to fully understand the impact of lenalidomide on QoL and to validate observed responses [49].

Generally, therapies such as lenalidomide have been reserved for transfusion-dependent patients; however, there are ongoing investigations exploring the possible benefits of starting treatments prior to transfusion dependence. An interim analysis of the phase 3 European Sintra-REV trial comparing lenalidomide to placebo in patients with non-transfusion-dependent del(5q) LR-MDS [52] showed that the patients receiving lenalidomide had a significantly longer time to transfusion dependence compared with patients receiving placebo (76 vs. 26 months; *P* = 0.021) [52]. However, a comparison to ESA would have been more in line with the current European guidelines.

For patients with non-del(5q) LR-MDS, there is less consensus on therapy options after the disease progresses while on ESA treatment or for those patients who are unlikely to respond to ESAs.

Responses to ESAs in combination with granulocyte colony-stimulating factors have been reported in specific subgroups, revealing an option for patients with insufficient ESA response, with the understanding that efficacy may be limited [53]. Additionally, two phase 3 trials have shown synergistic activity of epoetin alfa combined with lenalidomide compared with lenalidomide alone in patients without del(5q) who were not eligible for or were refractory to ESA [54, 55].

Another option for patients with MDS, particularly with RS—generally associated with mutations in *SF3B1*—who experience disease progression with ESA treatment, is luspatercept, which targets pathways associated with TGF-β signaling and enhances late-stage erythroid maturation [16]. ESMO 2021 and NCCN Guidelines® incorporate the use of luspatercept recognizing that patients with RS or *SF3B1* mutations appear more likely to respond to this treatment [14, 16, 22]. The U.S. Food and Drug Administration (FDA) and European Medicines Agency (EMA) have approved luspatercept for the treatment of patients following ESA therapy, particularly those with MDS with RS [16], as well as those with MDS/MPN with RS and thrombocytosis [56]. QoL was similar between patients receiving luspatercept and those receiving a placebo, despite a reduction in RBC transfusions, suggesting further work is needed to understand the impact of luspatercept on the patient experience [57]. The efficacy and safety of luspatercept is currently being compared to epoetin alfa in the ongoing phase 3 COMMANDS trial (NCT03682536), in RBC transfusion-dependent, ESA-naïve patients with LR-MDS with or without RS [58], and real-world experiences of MDS treatment with luspatercept are emerging. One study, which retrospectively evaluated luspatercept in MDS-RS patients in routine clinical practice, found limited value in securing durable anemia responses [59], while a single institution case series demonstrated potential clinical benefit in patients with LR-MDS with RS and *SF3B1* mutation [60]. It needs to be noted however, that reported adverse events of bone pain and arthralgia warranted dose reduction or treatment suspension in some cases [60]. In other studies, thromboembolic events, and high blood pressure in patients with MDS and β-thalassemia treated with luspatercept have also been reported [61]. Further understanding of the long-term impact of luspatercept on patients with MDS, including the cost-effectiveness of this agent, remains to be addressed.

Patients’ dependence on regular RBC transfusions may lead to progressive iron overload, which can eventually affect multiple organs (i.e., liver, heart, and endocrine organs) and is known to reduce survival in hereditary transfusion-dependent anemias [62–64]. Event-free survival (EFS), iron overload, and safety of iron chelation therapy (ICT) with deferasirox were evaluated in patients with IPSS Low- and Intermediate-1 risk MDS in the randomized TELESTO trial [65]. The group receiving ICT showed superior EFS compared with the placebo; however, due to reduced patient enrollment (210 instead of 630), was insufficiently powered to answer the question of whether there was a survival benefit to ICT in MDS [65]. Multiple studies indicate an impact on other clinical endpoints, including cardiac and hepatic, marrow failure, and infections [47]. Guidelines recommend considering ICT for adults with serum ferritin levels >1000 µg/L, receiving >15–75 RBC units, and candidates for allogeneic HSCT, recognizing that preference should be given to minimizing iron overload by improving transfusion requirements with MDS medications where possible [15, 16, 47, 62, 63]. Potential side effects of current ICT must also be considered, including renal insufficiency and gastrointestinal disturbances (deferasirox), injection site reactions, ophthalmologic/ototoxicity (deferoxamine), and a risk of agranulocytosis with deferiprone (not approved by health authorities for this patient group in many jurisdictions) [47].

In patients with LR-MDS with anemia and other severe cytopenias, the selection of second-line therapies varies according to the mutation profile, specific cytopenias present, and blast counts. The hypomethylating agents (HMAs), azacitidine or decitabine, may be considered, but are often reserved for later lines of therapy unless another indication is present (e.g., excess blasts or evolution to higher-risk features) [66]. Limited efficacy and suboptimal trial design (i.e., poor patient selection, underdosing of one treatment arm in a trial comparing two HMAs) are important caveats to interpretation of data on HMAs in LR-MDS [67].

Highlighting the significance of a comprehensive MDS evaluation, to the identification of patients with MDS-h, a disease entity that may have some overlapping features with aplastic anemia [15], remains important. Indeed, it is now proposed as a distinct MDS subgroup in the WHO 2022 classification [12]. In these patients with LR-MDS, refractory cytopenia, and hypoplastic bone marrow (<25% cellularity), we consider immune-suppressive treatment with anti-thymocyte globulin (ATG), cyclosporine with or without thrombopoietin-receptor agonist (TPO-RA), analogous to the treatment of aplastic anemia [15, 68]. In patients with symptomatic thrombocytopenia, we may consider TPO-RAs, azacitidine, or androgens (Fig. 2). For patients with symptomatic neutropenia, treatments may include HMA or growth factor support at times of infections (Fig. 2).

Other factors guiding treatment selection may include age, patient-based risk factors, treatment goals, RBC transfusion dependence, lack or loss of response to first-line treatment, fibrosis, and somatic mutations [16]. Importantly, as none of these chemotherapeutic approaches are curative, patient participation in a clinical trial should be considered at any stage of treatment. Finally, allogenic HSCT may be considered for select patients with LR-MDS, particularly if they are young, failed multiple lines of therapy or treatment with HMAs, or if they present with higher-risk molecular features [69]. Notably, there are ongoing efforts to understand whether the new prognostic models (e.g., IPSS-M) can effectively risk stratifying patients with LR-MDS with high-risk features in order to recommend the most beneficial treatment options, including an allogeneic HSCT [70].

### Assessment of response to treatment of LR-MDS

Historically, responses most relevant to LR-MDS included durable achievement of hematologic improvement (HI) or RBC-transfusion independence (RBC-TI), e.g., lasting ≥8 weeks. More recently, it is also recognized that response expectations may vary according to disease burden at the time of treatment initiation. For instance, in patients with high RBC transfusion burden (a receipt of ≥8 RBC U/16 weeks in ≥2 episodes), a 50% decrease in transfusions may be clinically meaningful, while for patients with lower RBC requirement at baseline (receipt of 3–7 RBC U/16 weeks in ≥2 episodes), achieving RBC-TI and improving baseline hemoglobin levels may be more meaningful. These considerations have led to a proposal for revisions to the IWG criteria for response assessment in LR-MDS, specifically pertaining to anemia and RBC transfusion needs [71]. These include defining a pre-treatment screening period of 16 weeks, dividing patients into three transfusion burden categories (non-transfused, low, and high transfusion burden), and an observation period of ≥16 weeks from treatment initiation for response assessment [71]. Improvement of QoL is relevant for patients with LR-MDS, and several PRO instruments focusing on QoL have been applied to patients with MDS [72]; however, there are limitations to the application of PROs, such as when they are administered and temporal events around their assessment (e.g., prior to or following transfusions). Additionally, the choice of instrument, frequency, and how this information should be applied to patient management, remains controversial [17]. Therefore, prospective assessment of standardized PROs in daily clinical care, including novel metric trackers (e.g., wearables), is urgently needed. The inclusion of PRO endpoints should be considered for future clinical trial design.

## Emerging treatments for patients with LR-MDS

Numerous novel targets, which promise to change the LR-MDS treatment landscape, have recently been identified. Current studies in LR-MDS are outlined in Table 4, while specific therapeutics are outlined below.

**Table 4:** Potential novel and emerging treatments (or indications) for patients with MDS.

Treatment	Currently used in	Key LR-MDS population inclusion/exclusion criteria
Lenalidomide	TD del(5q) LR-MDS [52] R/R or ineligible for ESA^a^	ESA-naïve, TI del(5q) LR-MDS [52]
Luspatercept	Non-del(5q) LR-MDS-RS, R/R or ineligible for ESA [16, 22]	• Non-del(5q) LR-MDS-RS and thrombocytosis, R/R or ineligible for ESA [56] • ESA-naïve, non-del(5q) LR-MDS with or without RS [58] [NCT03682536]
Imetelstat	–	LR-MDS, R/R or ineligible for ESA [73] [NCT02598661]
Roxadustat	Anemia in CKD in China [76] [NCT03263091]	ESA-naïve, non-del(5q) LR-MDS, preferentially without RS [77] [NCT03263091]
H3B-8800	–	MDS patients with *SF3B1, SRSF2*, or *U2AF1* mutations [80] [NCT02841540]
CC-486 (oral azacitidine)	Injectable azacitidine for LR- and HR-MDS [16, 22]	Oral azacitidine for LR- and HR-MDS [81] [NCT01566695, NCT02103478]
Nivolumab	Various malignancies [92]	MDS, in combination with azacitidine [93] [NCT02530463]
Ipilimumab	Melanoma among other malignancies [94]	MDS, in combination with azacitidine [93] [NCT02530463]

^a^According to ELN guidelines.

Abbreviations: *CKD* chronic kidney disease, *ELN* European LeukemiaNet, *ESA* erythropoiesis-stimulating agent, *HR-MDS* higher-risk MDS, *LR-MDS* lower-risk MDS, *LR-MDS-RS* lower-risk MDS with ring sideroblasts, *MDS* myelodysplastic syndromes, *R/R* relapsed/refractory, *RS* ring sideroblasts, *TD* transfusion dependent, *TI* transfusion independent.

### Imetelstat

Imetelstat is a first-in-class competitive inhibitor of telomerase enzymatic activity. In the phase 2 part of the phase 2/3 IMerge study (NCT02598661), patients with LR-MDS refractory to, or ineligible for ESA treatment and with a high transfusion burden (≥4 RBC U/8 weeks), received intravenous imetelstat at a 7.5 mg/kg dose in a 2-h infusion every 4 weeks until disease progression. Overall, 37% of patients achieved the primary endpoint (RBC-TI for ≥8 weeks) [73]. The RBC-TI response was shown to be durable, with 42%, 32%, and 29% of patients achieving RBC-TI ≥ 8 weeks, ≥24 weeks, and ≥52 weeks, respectively [74]. The median and maximum RBC-TI durations were 20 months and 2.7 years, respectively [74]. A reduction in cytogenetic and mutational malignant clonal burden was observed in some patients, suggesting imetelstat’s disease-modifying activity [73], although further study is needed. The phase 3 part of the IMerge trial, comparing the efficacy of imetelstat versus placebo, has recently reached the recruitment target, and results are anticipated [75].

### Roxadustat

Roxadustat is a hypoxia-inducible factor prolyl hydroxylase inhibitor approved in China for the treatment of anemia in patients with chronic kidney disease [76]. In a phase 3 study (NCT03263091), patients with non-del(5q) LR-MDS with <5% bone marrow blasts and low RBC transfusion burden (1–4 RBC U/8 weeks) received roxadustat (1.5, 2.0, or 2.5 mg/kg) orally three times weekly [77]. Roxadustat treatment resulted in RBC-TI lasting ≥56 consecutive days during the first 28 weeks of treatment in 37.5% of patients, while 54.2% achieved a ≥50% reduction in RBC transfusions [77]. At 1-year follow-up, the proportion of patients achieving RBC-TI ≥56 consecutive days remained at 37.5%, while the proportion of patients achieving a ≥50% reduction in RBC transfusions increased to 58.3% [77]. Subgroup analyses suggested that fewer patients with RS achieved RBC-TI for ≥56 consecutive days (23% vs. 55%), while baseline EPO had little effect on response (≤200 IU/L: 39%; 200–400 IU/L: 33%), although the sample size was small [77]. Notably, the FDA did not approve roxadustat for the treatment of anemia due to chronic kidney disease over concerns of increased risk of thrombotic and cardiovascular events [78].

### Spliceosome modulators

Dysplasia-defining splicing factor mutations (e.g., *SF3B1*, *SRSF2*, and *U2AF1*) are found in over half of MDS patients and, therefore, are an appealing therapeutic target. Moreover, they tend to be early mutational events, and are mutually exclusive, suggesting that MDS cells do not tolerate multiple alterations in critical splicing factor proteins. H3B-8800, an orally available small molecule modulator of *SF3B1*, induced synthetic lethality in spliceosome-mutant cancer models [79]. It was tested in 84 patients with myeloid cancers (42 with HR-MDS or LR-MDS; 88% with spliceosome mutations of interest; NCT02841540) [80] and 14% of patients experienced reduced transfusion requirement (RBC or platelets), although marrow responses and changes in mutation burden were not seen [80]. Splicing modulators, or other targets essential to pre-mRNA splicing, such as protein arginine methyltransferase 5 (PRMT5) or ataxia telangiectasia and Rad3-related protein (ATR), are being actively investigated.

### Oral HMAs

HMAs, or DNA methylation inhibitors (DNMTis), are used to treat patients with HR-MDS [14, 16, 22]. Important use limitations include the burden of treatment administration (subcutaneous) and local reactions, particularly in patients with LR-MDS for whom the burden of clinic visits relative to disease burden should be considered. However, oral administration of HMAs can allow for more flexible dosing and maintenance of patients’ autonomy. A phase 3 study of oral azacitidine (CC-486) versus placebo in patients with LR-MDS (NCT01566695) reported that 31% and 11% of patients, respectively, achieved the primary endpoint of RBC-TI ≥56 days [81]. Importantly, different formulations of azacitidine (oral vs. intravenous or subcutaneous) can have different pharmacokinetics, limiting them from being interchangeable, and different potential side effects, such as diarrhea with oral azacitidine (CC-586) and constipation with subcutaneous/intravenous azacitidine and its associated antiemetic regimens.

Combining oral cytidine deaminase inhibitors (e.g., cedazuridine) with oral DNMTi therapy allows for improved pharmacokinetics, similar to standard subcutaneous or intravenous DNMTi formulations. A combination of oral decitabine plus cedazuridine (ASTX727) was approved by the FDA for patients with IPSS Intermediate-1-, Intermediate-2-, and high-risk MDS, or chronic myelomonocytic leukemia, based on studies showing equivalence to intravenously administered decitabine (NCT02103478) [82]. A phase 1/2 study is currently evaluating the safety, pharmacodynamics, pharmacokinetics, and hematologic response to ASTX727 in patients with LR-MDS (NCT03502668). In phase 1 dose-escalation study in LR-MDS, a combination of oral azacitidine with cedazuridine (ASTX030) is also being assessed for equivalence with standard 7-day intravenous or subcutaneous azacitidine dosing (NCT04608110).

### Immune-based therapies and inflammatory pathways in LR-MDS

Increasing evidence indicates that the pathogenesis and progression of MDS are influenced by immune mechanisms, suggesting that treatments that modulate the responses of innate and adaptive immunity by targeting immune checkpoints, tumor antigens (vaccines), and the inflammasome may be active [83, 84].

Allogeneic HSCT remains the only known curative approach for many myeloid malignancies, including MDS, thought in part related to a “graft-versus-leukemia” effect from immune mediator cells [85]. Novel approaches using immuno-oncology targets are being explored in MDS, either as monotherapy or in combination with azacitidine [86]. However, any immunotherapy approach will likely need to be more nuanced in MDS; for instance, inflammatory pathways have also been implicated in the progression and maintenance of clonal hematopoiesis and the disease context might be crucial for any therapeutics in this space [87, 88].

Tumor vaccines are promising with the hope of inducing an anti-tumor immune response in patients with LR-MDS. A pilot trial of the K562/GM-CSF (GVAX) vaccine in five patients with MDS (three with LR-MDS), reported a reduced transfusion requirement in one patient, and HI in another [89]. Further exploration of tumor vaccines, perhaps incorporating novel targets (e.g., mutation-specific moieties) or design (e.g., patient-specific mRNA vaccines) may lead to novel future treatments for MDS.

Finally, an increased understanding of MDS pathogenesis suggests a role of the Nod-like receptor (NLR) family pyrin domain containing 3 (NLRP3) inflammasome activation, leading to cell death; NLRP3 inhibitors are in clinical development for LR-MDS treatment [84].

## Conclusions and future directions

Management of patients with LR-MDS is increasingly nuanced, due to the heterogeneity of patient- and disease-based factors, and the expanding number of approved treatment options or combinations available. These patients will typically live with MDS for >3 years, and decisions around therapy depend on the burden of disease, symptomatic complications, mutational profile, and overall goals of therapy. The most common cytopenia in LR-MDS is anemia, but its degree and clinical impact on potential comorbid conditions vary. Several therapies are currently available for the treatment of patients with anemia due to MDS, and more are being evaluated, making an optimal selection of therapies and the sequence of interventions, more relevant to patient management. Increasingly, the use of NGS has refined prognostication and sometimes offers targeted therapeutic options. In the future, mutational profiles may be incorporated into risk stratification schemes and treatment algorithms, resulting in a more targeted treatment approach.

Notably, over the last 20 years, the number of clinical trials initiated for LR-MDS treatments has remained limited [90]. Furthermore, few agents are being developed specifically for MDS; many phase 1 trials investigate one drug for other cancers and may include MDS only as a subset of the study. Given the particularities around MDS management and response, such as the emergence of treatment-related cytopenias [91], exploration of novel therapeutics in MDS during early testing phases may be limited. To increase the number of potential treatment options and to maximize their chances for successful clinical development, factors, including the patient population characteristics, specific molecular targets and/or pathways involved in MDS pathology, and revision of relevant endpoints, need to be considered [90]. Improvement and standardization of molecular response criteria and PRO assessments will be fundamental for the development of new, effective, and tolerable therapies for LR-MDS. Although there are more potential therapies available than before, the progress remains slow. That said, there are reasons for optimism; our increasing understanding of MDS-associated molecular pathways, and a more refined understanding of clinically meaningful trial endpoints, suggest tangible ways to achieve improved clinical outcomes in LR-MDS patients in the near future.

## Supplementary information

Supplementary material

## Data Availability

No datasets were generated or analyzed for this review paper.
