# Supplementary material for: Management of patients with lower-risk myelodysplastic syndromes
Source: Blood Cancer J. 2022 Dec 14;12(12):166. doi: 10.1038/s41408-022-00765-8 (PMC9751093; doi:10.1038/s41408-022-00765-8)
Supplement: Supplementary file 1 — Supplementary material [file 41408_2022_765_MOESM1_ESM.docx]

**SUPPLEMENTARY MATERIAL**

**Table S1** **Recommended flow cytometry immunophenotyping markers for assessment of MDS [1**–**5].**

|  | **Myeloid and lymphoid progenitor** | **Granulocyte** | **Monocyte** | **Erythroid lineage** |
| --- | --- | --- | --- | --- |
| **Core immunophenotyping markers** | **CD45, CD34, CD117,  HLA-DR** | **CD45**, **CD117** | **CD45** | **CD45,** **CD34, CD117** |
| **Recommended immunophenotyping markers** | CD13, CD33, CD10, CD11b, CD15, CD38, **CD7**, **CD56** | **HLA-DR**, **CD10**, **CD11b**, **CD13**, **CD16**, CD15, CD33, CD14, CD64, CD56 | **HLA-DR**, CD13, CD33, CD11b, **CD14,** **CD34,** **CD36/CD35^a^**, **CD64,** CD16, CD56, **CD117** | **HLA-DR,** **CD36,** **CD71,** **CD105,** CD13, **CD33** |
| **Optional immunophenotyping markers** | **TdT,** CD5, **CD19,** CD25, CD133, CD22 | CD5, CD7, **CD34** | CD2, MDC8 (Slan), **CD300e (IREM2)** | CD235a |

Examples of 8-marker panels assessing various cell lineages (**granulocytes**, **monocytes**, **erythroid cells,** and **aberrant expression of lymphoid-associated markers**) for the diagnosis of MDS are presented.

^a^CD36 is a marker informative of the erythroid differentiation. As it has been included in the 8-marker panel for erythroid lineage assessment, it was replaced in the panel for monocytic lineage assessment by the CD35 marker.

Abbreviation: *MDS* myelodysplastic syndromes.

**Table S2 Diagnostic criteria for MDS, ICUS, IDUS, CHIP, and CCUS [6**–**9].**

|  | **MDS** | **CHIP** | **CCUS** | **ICUS** | **IDUS** |
| --- | --- | --- | --- | --- | --- |
| Cytopenia(s) | Yes (for at least 4 months, except if excess blasts) | No | Yes | Yes | No |
| Hemoglobin (g/dL) | <10 | ≥11 | <11  (for ≥4 months) | <11  (for ≥4 months) | ≥11 |
| Neutrophils (/µL) | <1800 | ≥1500 | <1500  (for ≥4 months) | <1500  (for ≥4 months) | ≥1500 |
| Thrombocytes (/µL) | <100 000  (for ≥6 months) | ≥100 000 | <100 000  (for ≥4 months) | <100 000  (for ≥4 months) | ≥100 000 |
| BM dysplasia | >10% in ≥1 cell lineage | <10% | <10% | <10% | >10% in ≥1 cell lineage |
| BM blasts | ≥5% | <5% | <5% | <5% | <5% |
| Mutations | ≥1 MDS-defining cytogenetic alteration | ≥1 MDS-related mutation^a^ | ≥1 MDS-related mutation^a^ | None or not assessed | None or not assessed |

^a^Clonality defined by mutation of myeloid disorder-associated genes (including particularly *DNMT3A*, *ASXL1*, *TET2*, *JAK2*, and *TP53* genes), with a VAF >2%.

Abbreviations: *BM* bone marrow, *CCUS* clonal cytopenias of uncertain significance, *CHIP* clonal hematopoiesis of indeterminate potential, *ICUS* idiopathic cytopenias of uncertain significance, *IDUS* idiopathic dysplasia of unknown significance, *MDS* myelodysplastic syndromes, *VAF* variant allele frequency.

**Supplementary references**

1. van der Velden VHJ, Preijers F, Johansson U, Westers TM, Dunlop A, Porwit A *et al*. Flow cytometric analysis of myelodysplasia: pre-analytical and technical issues-Recommendations from the European LeukemiaNet. *Cytometry B Clin Cytom*. 2021 Dec 11; doi: 10.1002/cyto.b.22046. Online ahead of print.
2. van de Loosdrecht AA, Kern W, Porwit A, Valent P, Kordasti S, Cremers E *et al*. Clinical application of flow cytometry in patients with unexplained cytopenia and suspected myelodysplastic syndrome: a report of the European LeukemiaNet International MDS-Flow Cytometry Working Group. Cytometry B Clin Cytom. 2021 Dec 13; doi: 10.1002/cyto.b.22044. Online ahead of print.
3. van Dongen JJM, Lhermitte L, Böttcher S, Almeida J, van der Velden VHJ, Flores-Montero J *et al*. EuroFlow antibody panels for standardized n-dimensional flow cytometric immunophenotyping of normal, reactive and malignant leukocytes. *Leukemia* 2012: **26:** 1908–1975.
4. Duetz C, Westers TM, van de Loosdrecht AA. Clinical implication of multi-parameter flow cytometry in myelodysplastic syndromes. *Pathobiology* 2019; **86:** 14–23.
5. Westers TM, Ireland R, Kern W, Alhan C, Balleisen JS, Bettelheim P *et al*. Standardization of flow cytometry in myelodysplastic syndromes: a report from an international consortium and the European LeukemiaNet Working Group. *Leukemia* 2012; **26:** 1730–1741.
6. Chanias I, Bonadies N. Current standard of care in patients with myelodysplastic syndromes and future perspectives. *Healthbook TIMES, Oncol Hematol* 2020; **6:** 10–22.
7. Fenaux P, Haase D, Santini V, Sanz GF, Platzbecker U, Mey U. Myelodysplastic syndromes: ESMO Clinical Practice Guidelines for diagnosis, treatment and follow-up. *Ann Oncol* 2021; **32:** 142–156.
8. Valent P, Orazi A, Steensma DP, Ebert BL, Haase D, Malcovati L *et al*. Proposed minimal diagnostic criteria for myelodysplastic syndromes (MDS) and potential pre-MDS conditions. *Oncotarget* 2017; **8:** 73483–73500.
9. Steensma DP, Bejar R, Jaiswal S, Lindsley RC, Sekeres MA, Hasserjian RP *et al*. Clonal hematopoiesis of indeterminate potential and its distinction from myelodysplastic syndromes. *Blood* 2015; **126:** 9–16.
